# Cyber sickness in low-immersive, semi-immersive, and fully immersive virtual reality

**DOI:** 10.1007/s10055-021-00507-4

**Published:** 2021-05-19

**Authors:** Sergo Martirosov, Marek Bureš, Tomáš Zítka

**Affiliations:** University of West Bohemia, Univerzitní 8, Pilsen, 30100 Czech Republic

**Keywords:** Cyber sickness, Virtual reality, Stereoscopic projection, Head-mounted display, Dexterity test

## Abstract

It is known that virtual reality (VR) experience may cause cyber sickness. One aspect of VR is an immersion or otherwise sense of presence, the sense of feeling oneself in a virtual world. In this paper an experiment which was conducted in order to find the link between level of immersion and cyber sickness felt by participants is presented. Eighty-nine participants aged between 19 and 36 years have been equally divided into four groups with different level of VR immersion. The low-immersive group was represented by PC with monoscopic screen, the semi-immersive group was represented by CAVE with stereoscopic projector, the fully immersive group was represented by VR head-mounted display, and the last group was the control group without any kind of immersion. The task for the participants was to navigate through the maze for a specified amount of time (10 min). The Simulator Sickness Questionnaire was used as a subjective measure tool for cyber sickness level and Grooved Pegboard Test for assessing the fine dexterity, both before and after the experiment. Regarding the time spend in VR the fully immersive environment had the biggest problems as more than half of the participants had to stop before 10 min (*p* < 0.001). Concerning the cyber sickness, the significant increase in nausea score between pre-test and post-test scores has been observed in semi-immersive group (*p* = 0.0018) and fully immersive group (*p* < 0.0001). The increase in oculomotor score was smaller. The significant difference was noted only in fully immersive group (*p* = 0.0449). In spite of great nausea factor after the VR immersion the participants did not show a decrease of fine dexterity in any group (*p* < 0.001).

## Introduction

Nowadays virtual reality (VR) technology is becoming more and more popular. It is described as real-time interactive graphics with three-dimensional models, combined with a display technology that gives the user sense of the immersion in the model world and allows direct manipulation with objects in it (Bishop et al. 1992). As time goes by, many researchers try to find different use cases and benefits of virtual reality in various fields.

VR is widely used in medical area, where people train for various situations that they might face in real life (Lemole et al. 2007). Thanks to VR they can achieve higher skill and preparation, so that when the time comes to act, they would be ready to perform the actions correctly. This way, paramedics can perform training any time, with anyone and mostly everywhere (Schild et al. 2018). When it comes to human emotion and psychology, VR is also very helpful for managing human pain, anxiety, and other mental health issues. A research performed by (Prabhu et al. 2019) explores using biofeedback adaptive VR environment to reduce pain and anxiety of patients who have/are going to undergo surgical operation. The research focuses on utilizing affective computing techniques to develop and deliver an adaptive virtual reality experience based on the user's physiological response to reduce pain and anxiety and as the early results suggest, VR as a great potential in the management of pain and anxiety during pre- and postoperative care. Another common type of VR utilization is for flight simulations and trainings. Pilots train in VR simulation (Liu et al. 2018), in order to prepare themselves for various scenarios that can occur in real life, such as bad weather, broken engine. Professional pilots train mostly in special cockpits built for immersive/realistic training.

Many VR applications can be also seen in the light of events from the year 2020, when COVID-19 crisis broke out. VR proves to be an effective tool that can help handle this situation by spreading awareness, improving communication between people, and help in physical rehabilitation and pain management of an infected patient during the treatment process (Singh et al. 2020). Several previous studies like training how to properly keep hand hygiene to prevent infections (Clack et al. 2019), utilization of VR in online shopping with additional product visualization (Speicher 2018) or opportunities and challenges in online teaching and education with VR support (Callaghan et al. 2015) become very actual in 2020.

When it is impossible to perform training in real environment due to cost or complexity, VR training environments can be used to help in complex collaborative tasks (Moskaliuk et al. 2013a, b). When deciding whether to use VR or not, the DICE framework could help us. The acronym, coined by leading VR researcher and head of the Stanford Virtual Human Interaction Lab (VHIL) Jeremy Bailenson, stands for Dangerous, Impossible, Counterproductive, or Expensive and rare. In his book “Experience on Demand” (Bailenson 2018), Bailenson applies these indexes to discuss when the usage of VR is feasible or not. Moreover, VR training is often the only option available (Romano and Brna 2001). Also, it was noted that frequent opportunities for feedback, reflection, and revision, are important to improve the quality of learning (National Research Council 2000) and having a prepared VR training environment makes this possible.

A serious game or applied game is a game designed primarily for professional training rather than pure entertainment (Djaouti et al. 2011). Serious games tend to be used often in formal education and with sufficient support are shown to be highly motivational and effective in learning complex tasks (Chatti et al. 2012; Eseryel et al. 2014). Serious games are very popular for training and educational purposes. The trend shows increasing amount of publications and popularity on this subject. A study shows these findings and discusses both advantages and disadvantages of incorporating serious games into education and training (Zhonggen 2019). Virtual training environments (VTEs) keep learners motivated and engaged in learning process (Monahan et al. 2008) and have been proven very useful in pedagogical contexts (Mikropoulos and Natsis 2011). VTEs allow wide variety of learning styles and support collaborative knowledge building and reflective thinking (Mikropoulos and Natsis 2011) as well as experiential learning (Jarmon et al. 2009). Furthermore, some VR environments can support multiple user collaboration/cooperation, where students learn together and from each other (Kitchen and McDougall 1999).

As every other technology, VR has its own drawbacks. One of the significant is the cyber sickness (CS). As CS represents a considerable hurdle to using VR applications, much research has been conducted in this field in order to combat this problem. Some studies suggest systems that would predict the onset of CS even before occurring. This would be helpful as it is often better to predict and eliminate a problem before it occurs rather than face the consequences that would last longer to fix. There are some guidelines (Porcino et al. 2017) to using VR in order to minimize CS levels in head-mounted display systems. Some of those include field of view, duration, latency (Stauffert et al. 2018), acceleration, and navigation speed (Kwok et al. 2018). Further we will elaborate only those variables that are relevant with our proposed experiment.

Researchers believe that decreasing field of view tends to decrease VR sickness, though at the cost of sense of presence (Fernandes and Feiner 2016). As a result of increased field of view, users exhibited more postural instability, which has been suggested to be a surrogate measure for simulator sickness (Duh et al. 2001, 2002). In some research, it is noted that 3D video yields higher discomfort levels than 2D video (Kobayashi et al. 2015). It has been suggested that the size of the monitor and the resulting display angle can also influence the severity of virtually induced motion sickness (Duh et al. 2004; Emoto et al. 2008).

Often head-mounted devices are used for displaying virtual environments, and they are known to cause motion sickness (Patterson et al. 2006). A research conducted to analyze simulator sickness score using a video game in VR with head-mounted display (HMD), notes that sickness occurred in all participants who played the game for a maximum of 50 min (Merhi et al. 2007). Similar research suggests that sickness occurred both, in users who played a game on a standard TV screen and those with a HMD (Stoffregen et al. 2008).

It has been also noted that some users might exhibit symptoms of CS, both during and after VR experience (Bowman et al. 2001). CS is different from motion sickness in the way that the user is stationary, but has a compelling sense of motion as the visual imagery changes (Lathan 2001; Hale and Stanney 2002). Studies suggest that both exposure duration and repeated exposers are significantly linearly related to sickness outcomes (Kennedy et al. 2000). The repetitive watching of the same video image reduced subjective score of motion sickness in 8 of the 14 participants (Sugita et al. 2007). Research showed that women are more affected by CS than men. There is some controversy when it comes to age factor and CS, as some authors suggest that susceptibility to CS is greatest in younger ages; others mention the opposite (Arns and Cerney 2005; Brooks et al. 2010). People who are suffering from fatigue, sleep loss, upset stomach, and other types of illnesses are more susceptible to CS, as well as those with a history of migraines or concussion (Koslucher et al. 2016). It has been also noted that psychological conditions such as anxiety can significantly increase when simulated motion increases (Bruck and Watters 2009).

There is a wide variety of research conducted in the field of VR on simulator sickness: simulator sickness in different types of moving images (Kuze and Ukai 2008), comparison of one-screened and three-screened display types (Seay et al. 2001; Jinjakam and Hamamoto 2011), relationship between age and sickness level (Arns and Cerney 2005; Brooks et al. 2010), gender effect on simulator sickness (Parsons et al. 2004; Suma et al. 2010), etc.

The way how VR participants navigate and move in the VE (either by virtual or real walking) was explored in several studies. A study performed by (Suma et al. 2009) found that during the task requiring a navigationally complex environment and for a longer periods, it is preferable to use simulated walking. On the other hand, another study reported that natural walking caused less motion sickness than simulated walking in one out of two experiments involving a virtual maze (Chance et al. 1998). During a similar research, no differences were found in simulator sickness between real walking and several virtual travel techniques in a small virtual room (Zanbaka et al. 2005). It was also noted that more complex and more realistic scenarios caused higher levels of discomfort (Davis et al. 2015).

Simulation Sickness Questionnaire (SSQ) has been found to be valid and reliable subjective measure tool for both simulator sickness and side effects experienced in VEs (Hale and Stanney 2006; Kiryu and So 2007). It is interesting to note that the results of motion sickness after immersion in VE are much greater when both pre- and post-questionnaires are given rather than when only post-test questionnaire is used (Young et al. 2006). In several studies the SSQ was used as a tool for evaluation CS and comparison those results with machine learning algorithms. The results from study (Hell and Argyriou 2019) suggest that the proposed neural network architecture provides acceptable results in predicting motion sickness. As the time goes, the database would be extended more and more, which in turn would increase the reliability and precision of machine learning results. Another study on CS prediction suggests using an objective metric suck as postural instability in combination with Deep Long Short-Term Memory Model that was trained to specifically evaluate this metric during the experiment (Wang et al. 2019). The effectiveness of the proposed metric was analyzed and compared with subjective assessment methods based on SSQ which showed that the proposed method had the potential to be deployed within a closed-loop system and get real-time performance to predict VR sickness.

There are only few studies which focus and compare the influence of different levels of immersion on the CS. A study performed by (Papachristos et al. 2017) investigated the impact of two mainstream virtual reality headset technological approaches (oculus rift, a traditional HMD vs. a mobile-based VR headset) on the levels of spatial presence, usability, workload, simulator sickness, satisfaction and learning outcome in an educational virtual environment. The results, including simulation sickness, do not show differences in the variables studied, suggesting that mobile-based VR systems could provide acceptable levels of immersive user experience and contribute to the pedagogical use of VR. A study presented by (Polcar and Horejsi 2015) evaluated CS in different VE represented by PC, CAVE and Oculus Rift DK2. As a result the participants performing virtual tour with Oculus Rift DK2 were most affected by CS. In the study presented by (Kwok et al. 2018) the comparison of CS in CAVE and HTC Vive HMD was made. Similar to the previous study the HMD caused higher CS levels than the CAVE environment.

As a lot of VR simulations are designated for health sector the effect of VR on fine dexterity is worth exploring. For example, a study performed by (Collins et al. 2015) tested if the augmented reality version of Nine Hole Peg dexterity test would bring same results as the physical one. They found out that the current technologies are not yet reliable enough to capture real-life fine finger-level interactions for therapeutic purposes. Another study (Waliño-Paniagua et al. 2019) based on the similar principle elaborated if occupational therapy utilizing dexterity tests plus VR sessions supported by motion capture would speed up the process of convalescence. The Purdue Pegboard Test, the Jebsen-Taylor Hand Function Test, and the Grooved Pegboard Test were used for the physical assessment. No significant differences were not found in the manual dexterity between physical test performance and the performance backed by VR simulation; however, some improvements were found regarding the precision and effectiveness of certain functional tasks.

The literature review revealed that there is still a space for further elaboration of CS effects in different VE. Furthermore, although some studies that used dexterity tests have been found, those studies have not been using dexterity test as a tool for CS-level evaluation. Based on the previous studies we have proposed a laboratory experiment in order to map the level of CS in three VR environments. The low-immersive VR experiences are typically a desktop or laptop screens which present the VE to a user, so that the user does not experience the sense of actually being in the VE; the platform does not fully occlude the user's field of view. The semi-immersive experiences provide users with a partial VE to interact with. This type of VR is mainly used for educational and training purposes, and the experience is made possible with graphical computing and large projector systems. The fully immersive VR is a digital technology that allows users to experience artificial environments as the real world. In other words, users perceive virtual computer-generated surrounding using visuals, auditory, and haptics represented usually by head-mounted displays and gloves (Miller and Bugnariu 2016). The following research questions were formulated.

- Q1 What is the average time when users are experiencing some level of cyber sickness in different virtual reality immersions?
- Q2 What are cyber sickness level differences between low-immersive, semi-immersive, and fully immersive virtual reality?
- Q3 To what extent are the different immersions into virtual reality affecting the fine dexterity of the users?

## Methodology

This chapter describes the used methodology. We will explore in detail the experimental procedure, sample characteristics, and data collected.

### Experiment design

The experiment session consisted of pre-test questionnaires and dexterity test, maze experiment (in VE), and post-test questionnaire and dexterity test. The pre-test questionnaires were:

- Custom general questionnaire—contains information on age, sex, laterality an necessity of wearing glasses.
- Custom gaming questionnaire—information on 3D graphic software utilization, intensity of playing computer games (in hours per day), preferred console, and game controls.
- Standardized Simulation Sickness Questionnaire (Kennedy et al. 1993)—the questionnaire was translated to Czech by the research team. This questionnaire was filled also post-test.

Directly after finishing the filling of the questionnaires the participant performed the dexterity testing. For this purpose we used standardized Groove Pegboard Test (GPT). The test consists of a desk with 25 holes in which the pegs are being inserted. The pegs have a small stump which means that before insertion in the working desk, they need to be turned around precisely to fit in the holes (Bryden and Roy 2005). This test is thus very demanding on precise and fine handling of pegs which was the reason we selected it as the primary idea was that VR could affect this fine dexterity. There are two variants of the test. First variant evaluates the dominant hand, and second variant focuses on submissive hand. We tested only the dominant hand. Time needed to fill all holes with pegs was measured as an indicator (Ruff and Parker 1993). This dexterity test was also performed after VR exposure for comparison.

After finishing the dexterity test, the participant was moved to a room with specific virtual environment where he or she was exposed to it. There were three types of the virtual environment according to the level of immersion. For low-immersive VE we used personal computer (PC) with monoscopic screen, mouse and keyboard, semi-immersive VE was represented by CAVE (Cave Automatic Virtual Environment) with Intersense IS-900 for movement and for fully immersive VE the Oculus Rift CV1 with keyboard and mouse for movement was used. There was also one control group which was assigned by the task similar to the VR experiment in the real world. Immediately after spending 10 min in the virtual environment (or even less if they felt nausea) or in the real word (in case of control group), participant performed the post-test dexterity test and filled in the second Simulation Sickness Questionnaire. Besides the questionnaire data, the data from computers about the movement in VR, duration, etc., were obtained.

The whole process took approximately 30 to 35 min. All experiments were done during daylight time in the early autumn of 2018. The weather and light conditions were alike during the sessions. PC and Oculus condition had slightly dimmed window blinds to minimize unwanted screen reflections. CAVE room was dimmed significantly, with only little ambient light from several computer screens. No source of light shined directly at the participant except for the projection screen.

### Design of experimental groups

**Fig. 1:**
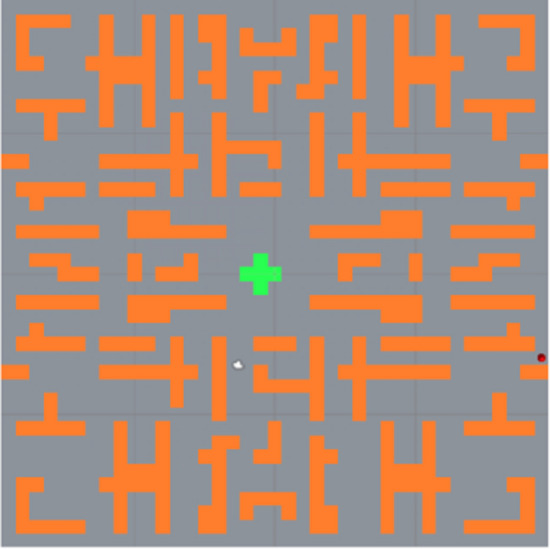
Maze map with spawned red ball (Color figure online)

**Fig. 2:**
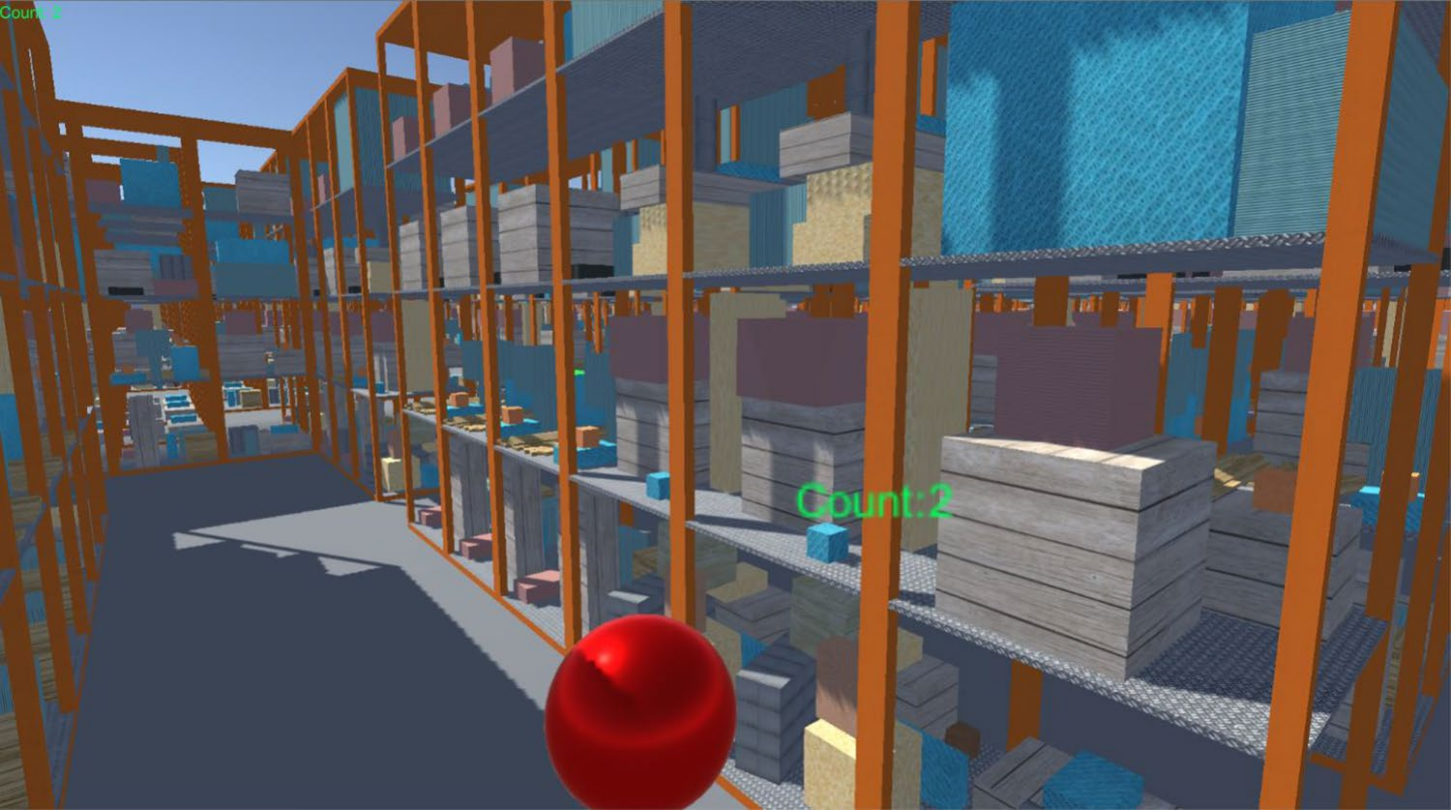
Screenshot of maze scene with the red ball (Color figure online)

The virtual environment for all three scenarios was in a form of custom-made PC game built in Unity3D 5.5.2 development environment. The game scene consisted of a randomly generated warehouse-like maze consisting of see-through warehouse shelves. The maze was subsequently edited manually to be symmetrical and to include large open area in the center of the maze. In this area, a green cross was placed on the floor (see Fig. 1) which was the starting point and the point of return. In the maze a floating red ball was randomly spawned. The goal of the participant was to find the red ball and pick it up by walking though it (see Fig. 2). Then a similar green ball was shown over the cross in the center of the maze, and participant had to return the red ball to the green one on the starting point, in order to count it to his or her score. First several red balls were placed near the starting area so that the participant could learn the task easier. The whole simulation was based on a real scenario in a warehouse where the storekeeper needs to find something in the right shelf and bring it back to a given place (like parts picking scenario). Participants of all three groups were being exposed for 10 min to this virtual environment, but some participants finished earlier due to cyber sickness symptoms occurrence.

### PC group

The PC group was exposed to the virtual environment in form of a PC application. The subject was seated in a comfortable office chair in front of the computer. They used a standard office-grade keyboard and mouse. The controlling was the same as any other first person computer game, with mouse for turning and keyboard for movement. The participants were free to use either the WSAD or the arrow keys. They could lower themselves (crouch) by pressing the Control key—this could have been used to see through the shelves. Computer setup had no problems sustaining a 60 FPS frame rate. The screen used was a Dell matte FHD 24″ IPS screen with 1920 × 1080 resolution at 60 Hz.

### CAVE group

**Fig. 3:**
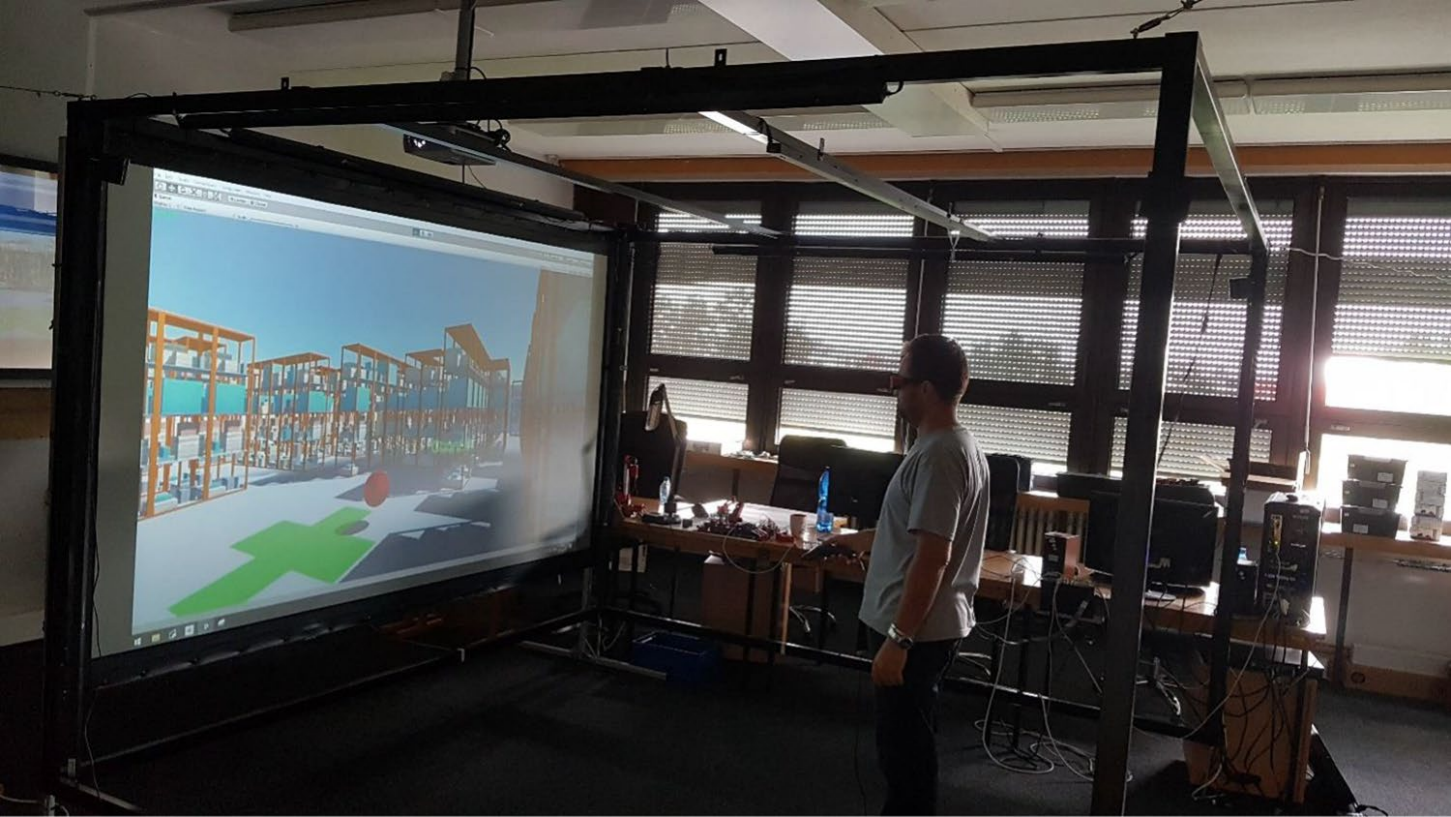
Setup of CAVE with participant performing the experiment (Color figure online)

The CAVE group was exposed to the virtual reality in a CAVE-like device, consisting of a single active 3D 300′’ back-projection screen with a DepthQ projector with a 1280 × 720 resolution and an Intersense IS-900 tracking device (see Fig. 3). The active stereoscopy glasses were Xpand3D X101 Infinity. On top of the glasses, there was a wired Intersense tracking sensor, allowing user to lean or crouch realistically (again this could be used to see through the shelves). The position of the tracking sensor in the frame was translated to camera’s local position in the player’s root coordinate system. The rotation of the sensor was not transferred to the camera, so that the view could remain still and the user could rotate the head himself. The participant was standing in the middle of the tracked area he could crouch physically. For movement, he used a wired version of Intersense Microtrax Wand flystick (controller). The wires were tethered together and hung from the Intersense carrier frame using a karabiner, which could be moved to tweak the cables so that their weight was as least uncomfortable to the participant as possible. The frame rate was kept at 60 FPS.

### Oculus rift group

**Fig. 4:**
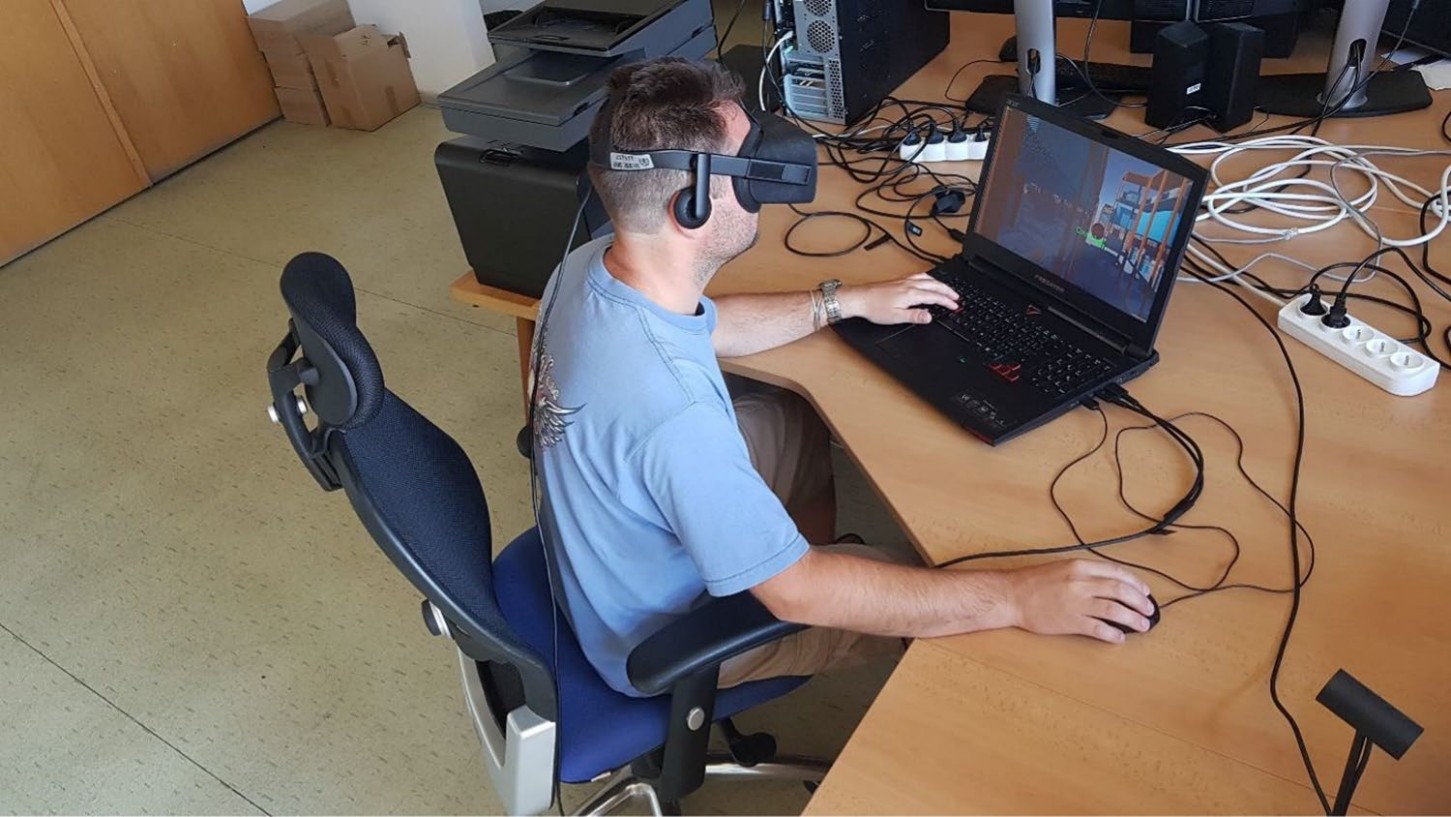
Setup of Oculus Rift CV1 with participant performing the experiment (Color figure online)

The Oculus group’s setup was almost the same as the PC group’s setup. The subjects were sitting in the same chair model as the PC group and were controlling the environment in the same way using mouse and keyboard instead of Oculus controllers which were not available. The Oculus Rift CV1 headset was used for looking around in all directions (see Fig. 4). Forward movement direction after the forward key press was in the direction of the view. The headset was connected to a laptop able of sustaining a high 75 FPS HMD’s native frame rate. Participants used the laptop keyboard and a standard office-grade mouse.

### Control group

**Fig. 5:**
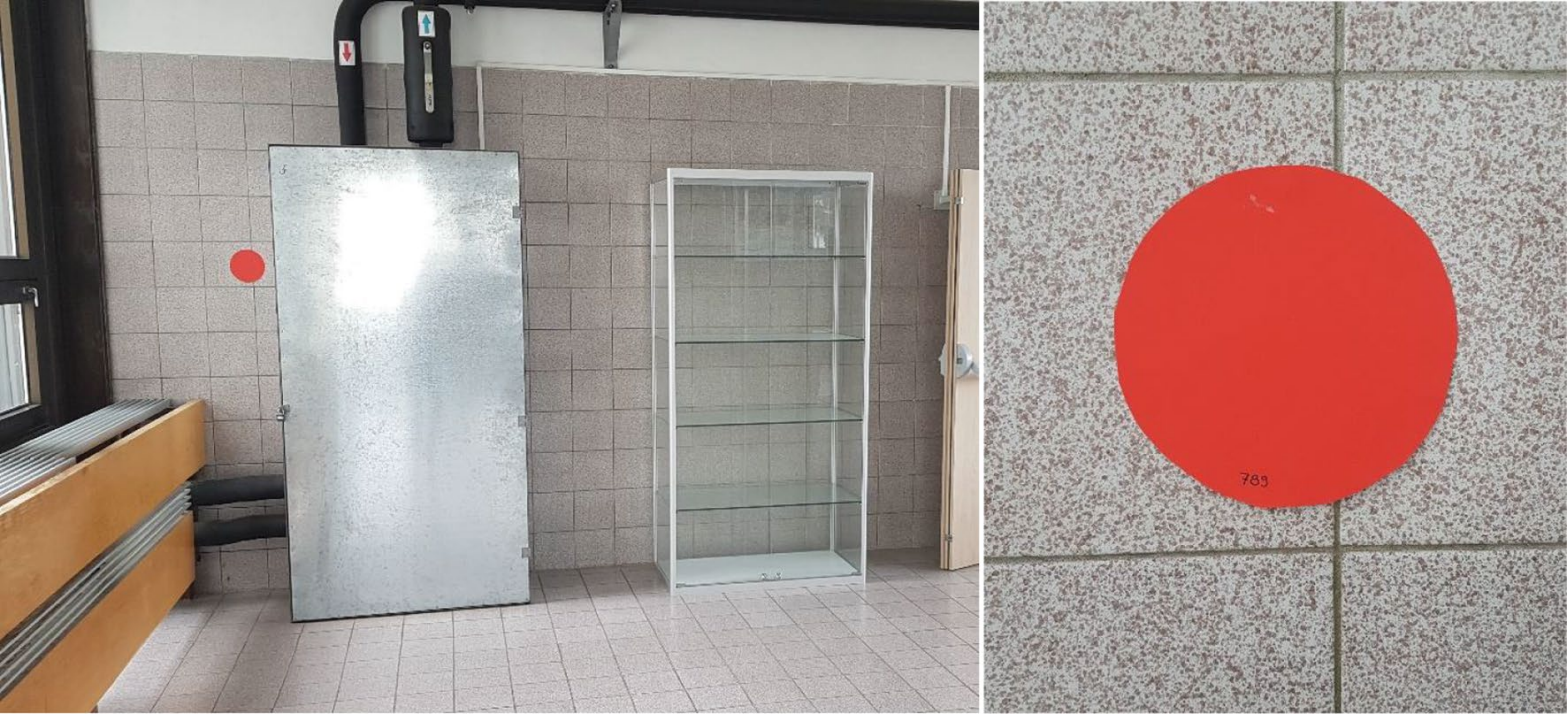
Example of red marker placement in control group experiment (Color figure online)

We have also included the control group in our experiment. The main reason for including the control group was to verify the dexterity tests. The participants in control group had the same time (10 min) to perform the task as the participants in previous VE groups. The task was of similar kind, to go through the university corridors and search for the red rounded markers. There were in total 15 markers attached mostly to walls as visualized in Fig. 5. The markers were partially hidden from some viewpoints, but there was no need to move furniture or other devices in order to see the marker. The participants were instructed to search for the markers and if found, note the number of the marker. The time was measured by stopwatches which they had with them. After 10 min the time signal informed them to return to the starting point.

### Participants

For the experiment, we drafted 83 students from Faculty of Mechanical Engineering, University of West Bohemia, in Pilsen along with six members of research center and faculty staff which makes 89 participants (67 males, 22 females) in total. Age of participants ranged from 19 to 36 (mean 22.65, median 22). Participants were randomly distributed into three groups corresponding to different VR types and one control group:

- PC group (monoscopic screen) consisted of 21 participants (15 males, 6 females). Age of participants in PC group ranged from 19 to 26 years, average 22.00 and median 22.
- CAVE group (stereoscopic projector) consisted of 23 participants (17 males, 6 females). Age of participants in CAVE group ranged from 20 to 31 years, average 22.95 and median 23.
- Oculus group (VR Headset) consisted of 24 participants (20 males, 4 females). Age of participants in Oculus group ranged from 20 to 36 years, average 23.42 and median 22.5.
- Control group (navigation in building) consisted of 21 participants (15 males, 6 females). Age of participants ranged from 20 to 29 years, average 22.10 and median 21.

It can be seen that age of participants did not differ significantly between groups. All participants signed their informed consent to take part in the experiments. The study was approved by the university Ethics Committee. Only two participants reported extended experience with immersive 3D VR display, and only three participants reported watching 3D TV regularly.

### Statistical analysis

For the statistical evaluation the Python language (version 3.7) and SciPy statistical package (version 1.1.0) were used. The obtained data were tested for normality by Pearson test in order to decide whether we should use parametric or nonparametric statistical tests. For data that were not normally distributed we used nonparametric Kruskal–Wallis test. The data with normal distribution were tested by appropriate parametric analysis of variance (ANOVA) either one-way or two-way ANOVA. We tested all hypotheses at level of significance *α* = *0.05*. All effect sizes are computed according to (Coe 2002) as pre-test vs post-test measurement with pre-test measurement taking role of a control group.

## Results

In the following chapter we have summarized the main results of our experiments. We have evaluated the cyber sickness level caused by different VE, behavior of participants in VR, and also the influence of VR on fine dexterity.

### Time spent in VR

**Fig. 6:**
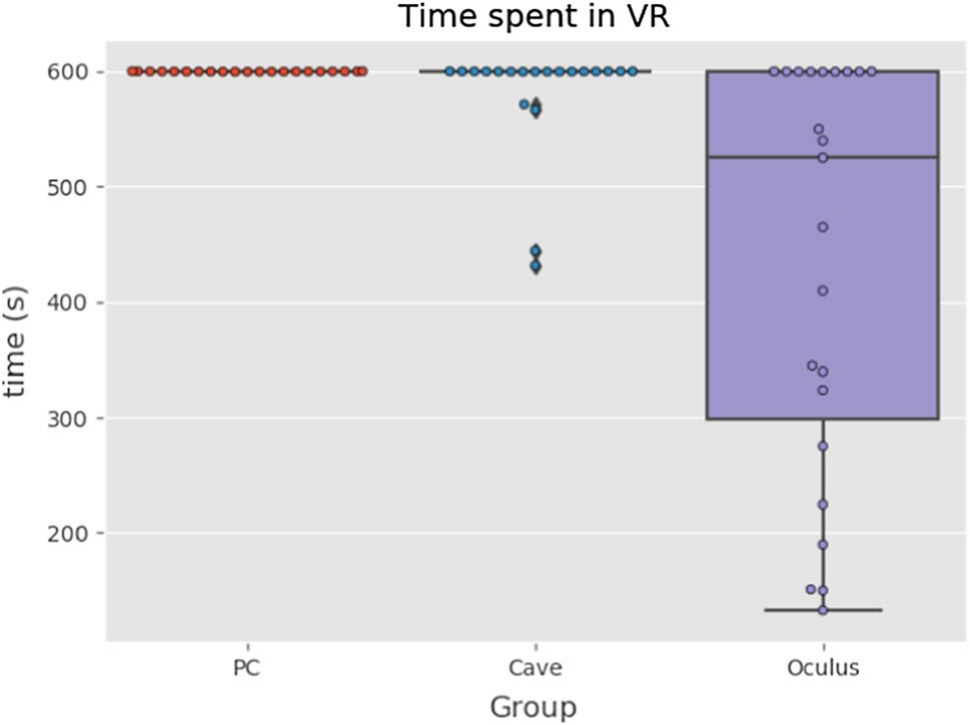
Time spent in different VR types (Color figure online)

Starting with time spent in VE simulation, Fig. 6 shows that all of the participants in PC group (on monoscopic screen) finished simulation with no difficulties. In CAVE (stereoscopic screen), some of the participants had to stop simulation sooner. When it comes to Oculus, we can clearly see that only less than half of participants could make it to the end. More than half had to stop before 10-min mark due to feeling cyber sick. This difference was statistically significant (*p* = *0.00027*). We have used nonparametrical Kruskal–Wallis test as the data did not have normal distribution. This discrepancy poses difficulties in analysis of other variables. Due to low number of participants we do not split them into groups by time and including time as factor in ANOVA gives unreliable results. For this reason we present exclusively the VR type as independent variable.

### Sickness scores

For the evaluation the Simulation Sickness Questionnaire was divided into two parts. The first part was focused on nausea where we considered factors like salivation increasing, sweating, nausea, dizziness, vertigo, stomach awareness, and burping. The second part was focused on oculomotor, considering factors like fatigue, headache, eye strain, difficulty in focusing or concentrating, fullness of the head, and blurred vision.

**Fig. 7:**
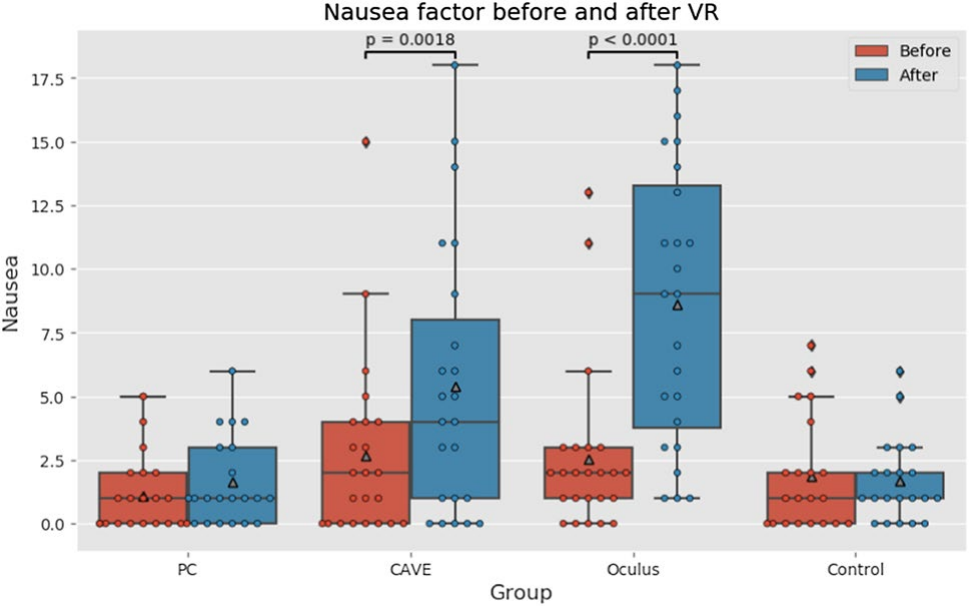
Nausea factor boxplots (Color figure online)

Regarding VR sickness score of nausea there were no significant differences between experimental groups in pre-test scores meaning we had homogenous sample which allows us to compare post-test scores. Kruskal–Wallis test (data did not have normal distribution) shows significant difference between post-test measurements (*p* < *10*^*–5*^). As boxplot in Fig. 7 shows, this difference came from CAVE and Oculus groups’ scores meaning that they affect nausea factor more than other two groups. The boxplots also suggest significant difference in pre-test and post-test scores in CAVE and Oculus group. This was confirmed by Wilcoxon paired tests (data did not have normal distribution) for both CAVE group (*p* = *0.00177*) and for Oculus group (*p* < *10*^*–4*^) and also their effect sizes 0.75 and 1.8. There was no perceivable change in control group.

**Fig. 8:**
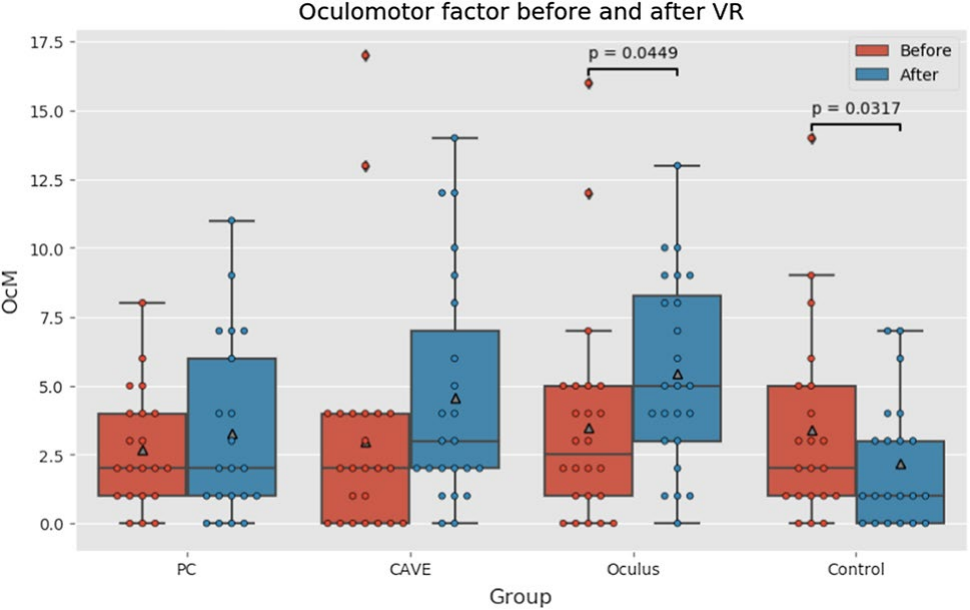
Oculomotor factor boxplots (Color figure online)

In oculomotor score (plotted in Fig. 8), there were no significant differences between groups in pre-test scores, suggesting again that we had homogenous group. In contrast with nausea factor, the effect is not as strongly supported by Kruskal–Wallis test (*p* = *0.00618)*. The differences between groups in post-test measurement were visibly smaller as seen in Fig. 5. This suggests that oculomotor factor is not affected as strongly as nausea factor by exposition to VR. In pre-test vs post-test scores Wilcoxon paired test shows significant difference in Oculus group (*p* = *0.0449)* with effect size 0.51 and in control group (p = 0.0317). For the control group the effect size is negative (-0.339), suggesting that brief period of walking slightly improved participants sense of coordination.

When time spent in VR is included as independent variable in ANOVA, there is very weak and small negative correlation between it and both sickness factors. This shows seemingly illogical dependency—the longer the time spent in VR the lower the sickness scores. This is, however, easily explained away by inverse dependency—participants who were experiencing strong cyber sickness had to quit the VR earlier.

### Behavior of participants in VR

Besides the data on cyber sickness and time spent in VR, we gathered various data on behavior of participant in the virtual environment, like number of stops, number of balls collected, or distance walked. For control group, these data are not available for obvious reasons and we omit it in this part of analysis.

**Fig. 9:**
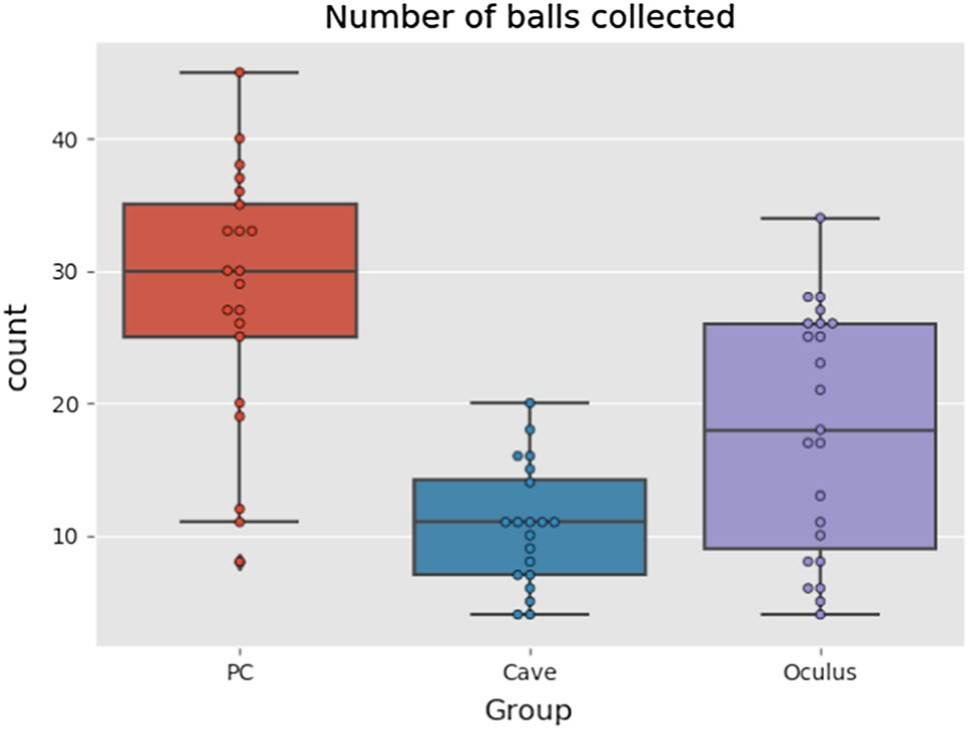
Boxplots of total number of collected balls (Color figure online)

The amount of balls participants collected overall, while they were in the VE as shown in Fig. 9. The participants from PC group collected the most balls, followed by Oculus group and CAVE group participants collected the least balls. It seems that the comfort of navigation in the VE was the main reason for the result. PC and Oculus group had the same controllers (keyboard and mouse). The only difference was in VR Headset. The CAVE used Intersense controller which was basically a joystick. We incorporated only one button from the joystick, assuming the difficulty of understanding the device should be low, but obviously it matters. Also the sensitivity of the joystick could be another factor.

The differences were statistically significant for all three groups (Kruskal–Wallis test, *p* < *10*^*–5*^) and also for Oculus vs. CAVE (Kruskal–Wallis test, *p* = *0.0128*). However, these data are hardly comparable as participants spent usually less time in Oculus environment, that is why we also evaluated the number of balls collected per minute.

**Fig. 10:**
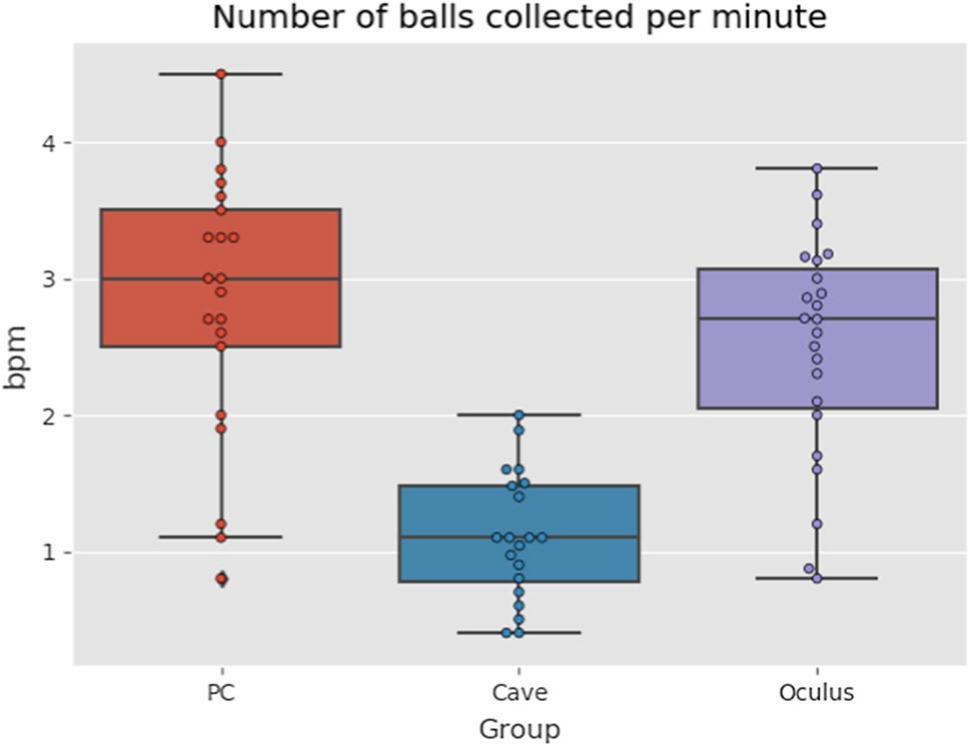
Boxplots of balls collected per minute (Color figure online)

**Fig. 11:**
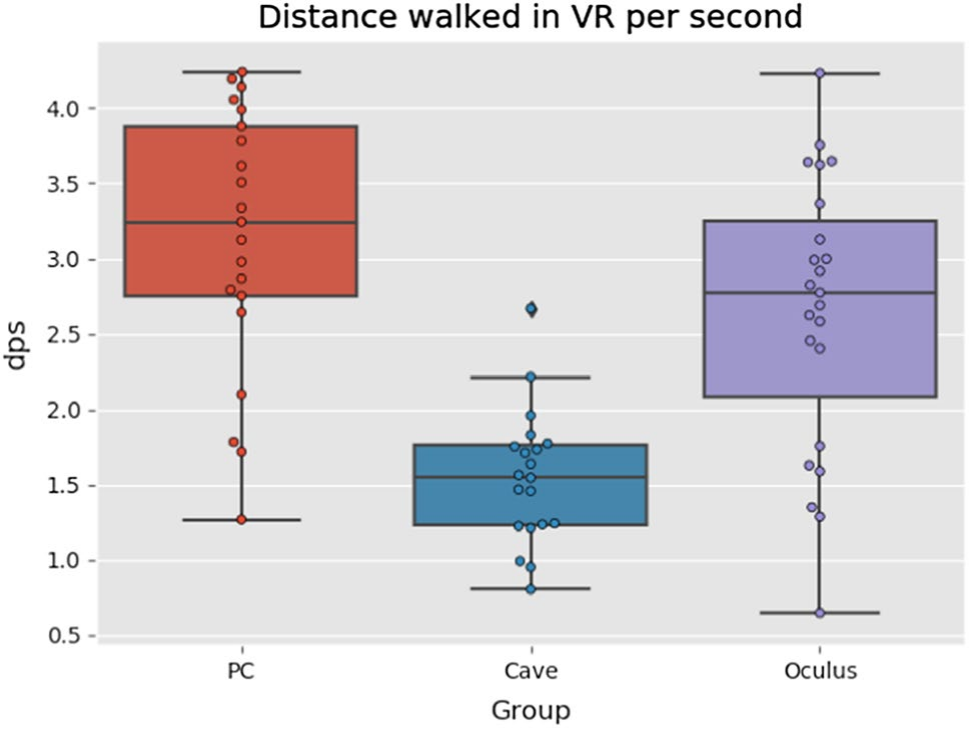
Distance walked per second boxplots (Color figure online)

The results of balls collected per minute (see Fig. 10) and distance walked per second (see Fig. 11) show similar trend. Highest amount of balls collected per minute was in PC group, followed by Oculus group and the last one was CAVE group. As the data had a normal distribution we used one-way ANOVA this time. The differences were statistically significant (*p* < *10*^*–8*^). However, the difference between Oculus and PC group was not statistically significant.

Accordingly, Fig. 11 clearly shows that the CAVE group walked the least amount. Again, the one-way ANOVA was used as the data had normal distribution. The differences were statistically significant (*p* < *10*^*–7*^). And again the difference between Oculus and PC group was not statistically significant. These results, however, do not necessarily imply that CAVE group suffered the most cyber sickness. The main reason could be the unfamiliar type of controller for the virtual environment.

### Fine dexterity

**Fig. 12:**
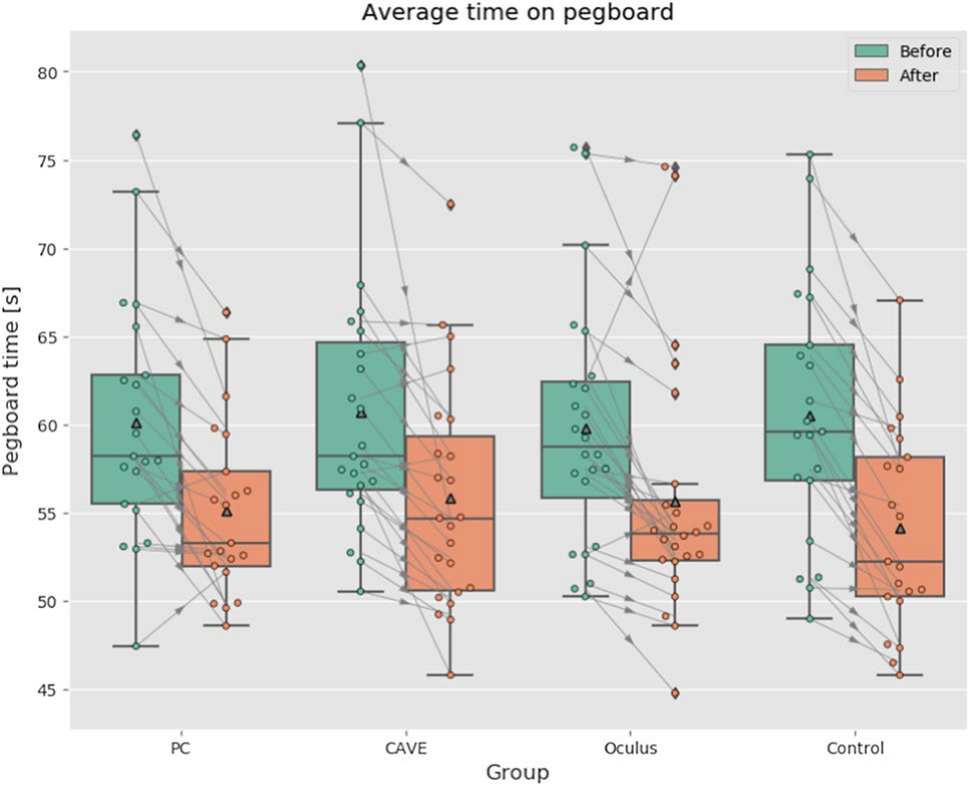
Results of dexterity test before and after the VE exposure (Color figure online)

Last but not least we have evaluated the results of dexterity tests performed before and after the immersion in the virtual environment. As Table 1 suggests there were no significant differences between experimental groups in pre-test scores, this was confirmed by Kruskal–Wallis test (*p* = *0.974*) which means we had homogenous sample. The mean time of the test before VR was around 60 s. This can be also seen in Fig. 12. There were no significant differences between groups in post-test scores either (Kruskal–Wallis test, *p* = *0.853).* The boxplots in Fig. 12 suggest significant difference—improvement in pre-test and post-test scores in all groups. This was confirmed by Wilcoxon paired tests (data did not have normal distribution) for all groups (*all p* < *0.001*) and very similar negative effect sizes (see Table 1). These differences mean that the participants improved in the time needed to complete the dexterity test regardless of VR exposition. Thus we conclude that the immersion in VR did not affect the participants regarding the fine dexterity.

**Table 1:** Results of dexterity tests

	Count	Before VR				After VR					
		Mean [sec.]	SD [sec.]	Min [sec.]	Max [sec.]	Mean [sec.]	SD [sec.]	Min [sec.]	Max [sec.]	p	Effect size
PC	21	60.15	6.91	47.45	76.40	55.16	4.94	48.6	66.35	0.0004	-0.72
CAVE	23	60.72	7.40	50.55	80.35	55.84	6.46	45.8	72.50	0.0001	-0.66
Oculus	24	59.83	6.97	50.25	75.70	55.67	7.23	44.8	74.60	5.9e-5	-0.60
Control	21	60.55	7.36	49.00	75.30	54.12	5.79	45.8	67.05	0.0005	-0.87

The connection lines in the boxplot diagram also suggest the progress of each participant. From this we can clearly see that nearly all participants improved in the time needed for test completion. There were only 2 participants in PC group, 3 participants in CAVE group, and 2 participants in Oculus group that performed worse after VR than before. Thus, only those several people were probably affected by VR exposure.

## Discussion

At the beginning of our research we have stated three research questions on which we have been searching for answers. In this discussion we will try to conclude on them.

Q1—What is the average time when users are experiencing some level of cyber sickness in different virtual reality immersions?

Although some participants (17%) in the CAVE group finished the experiment earlier than after 10 min, the biggest number of participants (58%) that finished earlier was noted in the Oculus group. The average time for exiting the experiment in this group was 7,25 min (median 8,73 min). Also, in this group they have reported highest scores of cyber sickness as described further.

Our participants quit the experiment even earlier than in the experiment reported by (Merhi et al. 2007). In this experiment the Xbox console and two types of games have been used to test the endurance of participants to cyber sickness. The Simulation Sickness Questionnaire was also used for measuring the sickness score. The participants were instructed to play the games for 50 min but were able to quite earlier if they experienced any symptoms of sickness. In standing position nearly 100% of the participants quitted after 16 min. In sitting position, 60% of the participants quitted in 14 min and further 12% of the participants quitted after 36 min. The rest lasted out till the end but also reported severe sickness score.

Q2—What are cyber sickness level differences between low-immersive, semi-immersive, and fully immersive virtual reality?

For clarification of the second research question we used evaluation by the Simulation Sickness Questionnaire. The score of nausea after the VR exposure was far higher in the CAVE group (*p* = *0.0018*) and even highest in the Oculus group (*p* < *0.0001*) which indicates that this environment made the participants sick. The most frequent symptoms were sweating, nausea, and dizziness. The oculomotor score after the VR exposure was not as high as in case of nausea score, but it was also increased as before the exposure. Again, the increase was noted mainly in Oculus group (*p* = *0.0449*).

The research presented by (Polcar and Horejsi 2015) was based on similar principles and obtained similar results. The scenario of the experiment was a 3-min virtual tour through the company. This virtual tour was performed as in our research on PC, CAVE and with Oculus Rift DK2. A total of 45 university students have participated in the experiment. The cyber sickness symptoms occurred between 13 students (29%) in CAVE environment and between 24 students (53%) with Oculus HMD. Unfortunately this research does not contain further detailed information on simulation sickness scores and intensity.

Another study presented by (Kwok et al. 2018) examined the effect of navigation speed as well as the use of different VR devices for navigation on cyber sickness. The scenario was in form of street navigation where 40 participants were standing still and automatically flying though with speeds either 10 m/s or 24 m/s. During the street navigation the participants were asked to count the occurrences of plastic bollards. The 4-wall CAVE system and HTC Vive HMD were the devices used for the VR. Each trial lasted for 5 min, and participants were asked to fill the Simulation Sickness questionnaire before and after the experiment. Results showed that higher navigation speed leads to increase in ratings of cyber sickness (*p* = *0.017*). The significant differences were also noted between devices. The CAVE participants reported lower sickness symptoms than the Oculus group (*p* = *0.05*).

Q3—To what extent are the different immersions into virtual reality affecting the fine dexterity of the users?

According to expected results from previous testing regarding the level of cyber sickness we also thought that the VR exposure might have influence on fine dexterity. That is why the participants performed the Grooved Pegboard Test before and immediately after the exposure. To our surprise the results of the dexterity test have not been affected. The participants improved in time needed for completion of the test as normally as without the VR exposure. Only several participants across all groups had worst results. Those were the most affected by the cyber sickness. We can say that similar results were observed in study (Waliño-Paniagua et al. 2019) where the participants also slightly improved the GPT time after the VR exposure. Although the studies cannot be easily compared as the Waliño-Paniagua study focused on patients with multiple sclerosis (our participants were completely healthy) and the VR environment was different than ours.

**Fig. 13:**
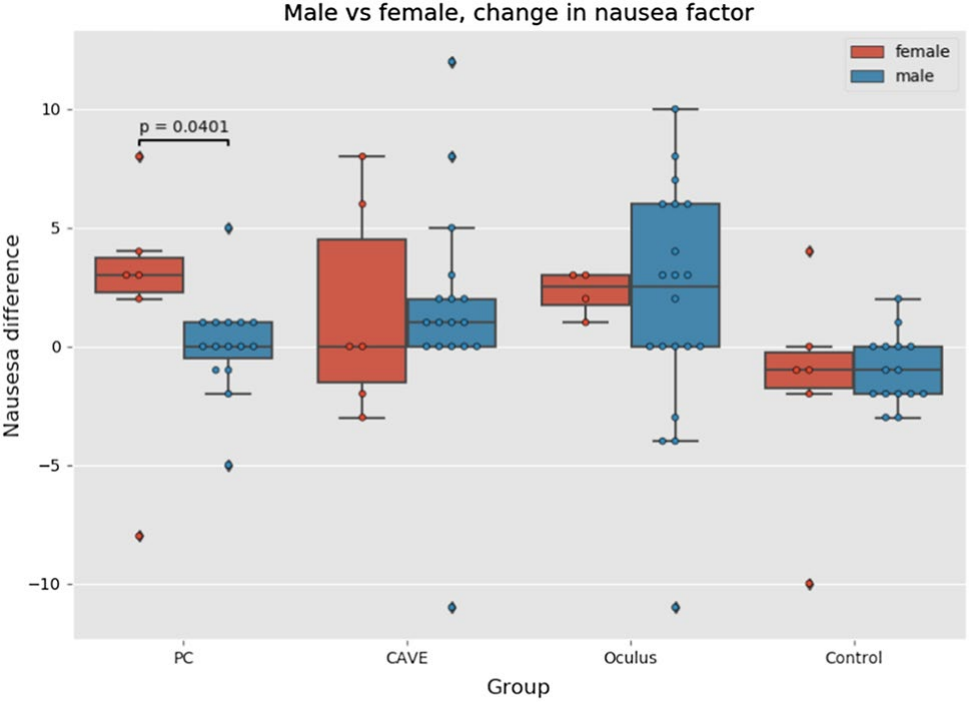
Comparison of nausea between genders (Color figure online)

Some previous studies like (Parsons et al. 2004; Suma et al. 2010) also focused on role of sex regarding the perception of virtual environments. While Parsons et al. (Parsons et al. 2004) reported that gender had no effect on rotational ability in the virtual environment, Koslucher (Koslucher et al. 2016) reported that females were more affected by motion sickness in their experiment. Unfortunately, the number of females (only 16 females) in our experiment was too small for making some qualified conclusions. Nevertheless, we tried at least to depict the results in Fig. 13. The Kruskal–Wallis test showed statistically significant difference only in PC group (*p* = *0.040)* which suggests that females were getting sicker than males. The differences in other VR groups were not statistically significant. Those results are however only approximate as we did not have enough female participants in individual groups to make the qualified conclusion.

**Fig. 14:**
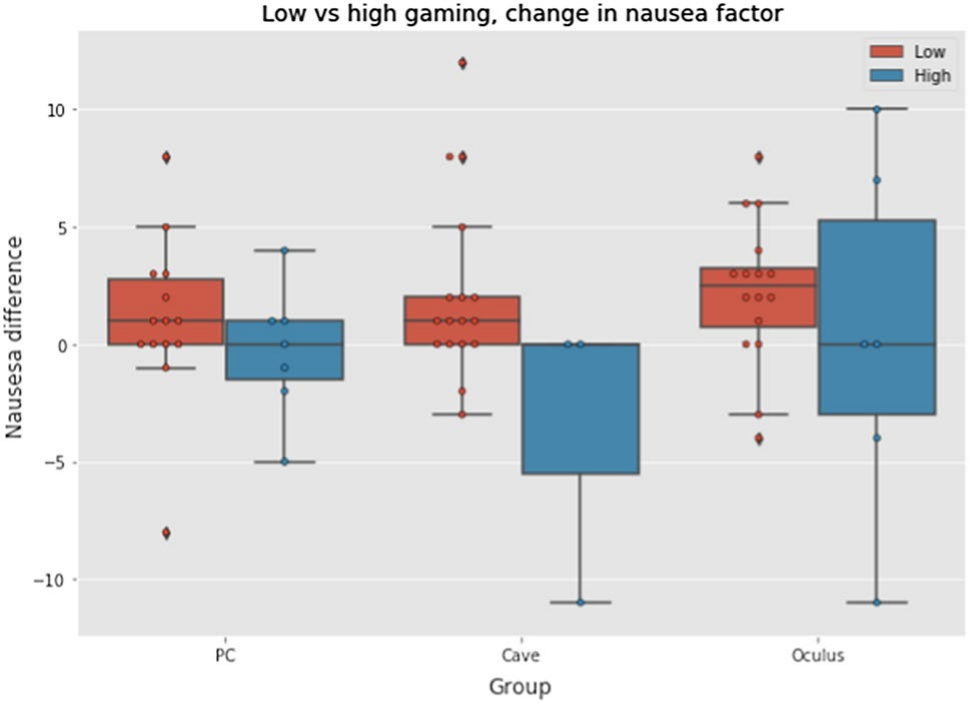
Comparison of nausea between gamers and nongamers (Color figure online)

In some studies like (Boot et al. 2008), the gaming experience is linked to higher focus, attention, and performance when compared to nongamers. The gaming questionnaire which was filled by the participants before the VR exposure was done in order to analyze also this effect. Excluding control group we had a sample of 45 gamers and 18 nongamers (5 participants have not been included as they filled the questionnaire incorrectly). The group of gamers was divided to high gamers (N = 16) who played more than five hours a week and group of low gamers (N = 29) who played less than five hours a week. It can be seen that the high gamers group was not very large, so again we could not perform any valid analyses. Anyway, similarly as in the case of genders, we tried at least to depict the results by a boxplots in Fig. 14. From this figure we can see that median values of nausea factor difference between pre-test and post-test questionnaire were higher in the group of low gamers in all VR types. Due to the small sample in each VR group, the boxplot whiskers are quite stretched and we cannot draw substantial conclusions. However, we can state and assumption that stronger gamers will be more resistant to cyber sickness.

There are many factors both technical and individual that could affect cyber sickness levels in users. Those factors, especially the technical, can be the subjects of further research. Some of the controlled aspects that could be adjusted and manipulated in some ways are frame rate, field of view, graphic resolution and bit rate of the visual output. A study performed by (Vasylevska et al. 2019) was specifically measuring the level of discomfort while showing different quality 360º videos. The study found that those parameters could have significant effect on comfort levels for users, and thus must be considered.

A type of movement in VR, which might be walking or teleporting, might be also a subject of further research. Some of already performed studies (Dorado and Figueroa 2015; Clifton and Palmisano 2020) suggests that there is no difference between teleportation technique in VR and walking/sliding that we used in our experiment regarding the level of cyber sickness. Others like (Bozgeyikli et al. 2016) suggest teleportation to be the most comfortable. From our own experience, the teleportation is the most comfortable type of movement but provides lesser feeling of presence in VR. Due to the nature of our VE, using teleportation technique in our experiment was not effective. If we are talking about movements type in VR we must not forget about speed. In order to improve and optimize the user experience the fast tracking of the head movement must be ensured. Reducing the effective latency (time lag) as described in (Lavalle et al. 2014) will positively affect the occurrence of cyber sickness. Last but not least the environmental factors (Bockelman and Lingum 2017) such as ambient noise, temperature, human factors, smell (Narciso et al. 2019), brightness (Vasylevska et al. 2019), could be taken into account when dealing with cyber sickness. However, by these factors it is very hard to predict the final outcome for each individual.

We have mentioned several quite important factors that are `influencing the cyber sickness level. There are already several ongoing researches on these topics, but as we saw similar studies can give different results. This fact leaves the door open for performing the same tests multiple times in order to make sure which results are the most common.

Based on results of our experiment and according to our experience we can propose the following utilization of different VR in practice. The fully immersive VR environments would be suitable for the cases where users will not need to move fast and where their view would not be obstructed with unnecessary 3D models, which creates visual noise. Industrial applications that are using VR for training, assemblies, or maintenance do not require fast movement, as some VR games might. Thus, using teleportation technique, higher-quality models, and lesser visual noise could aid in reduction for cyber sickness. As for the semi-immersive VR, for which the CAVE was used, the users would be able to partake much longer VR sessions. Additionally, CAVE allows cooperative VR sessions, where users do not have to take their stereoscopic glasses off to see each other and their surroundings. Low-immersive VR is just a PC with 2D monitor. Learning and training by this way existed for a long time and is mostly useful, although as research suggests, VR is more fun and enjoyable, thus more motivating.

## Conclusion

This paper focused on clarification of cyber sickness level in three different virtual reality environments low-immersive, semi-immersive, and fully immersive. As an assessment tool we used standardized Simulation Sickness Questionnaire and Grooved Pegboard Test for dexterity assessment. Both tools were applied before and after immersion into the virtual reality environment. We have also developed our own maze-like virtual environment in Unity3D where participants had to search for red balls and return them to starting position. This task was assigned for 10 min. As a result, more than half of the participants could not last those 10 min in fully immersive environment simulated by Oculus head-mounted display due to cyber sickness. Besides giving up earlier participants also reported severe nausea and oculomotor problems in Simulation Sickness Questionnaire. In semi-immersive environment represented by CAVE, cyber sickness was also reported but not to such an extent. Other statistics like number of collected balls or distance walked in virtual environment have been evaluated. Despite reported cyber sickness discomfort, it has not influenced the fine dexterity as the dexterity test performed after the immersion took less time to complete than before immersion. Thus, learning curve was not affected. Our paper confirmed that the level of cyber sickness is related to the type of virtual reality environment. In order to lower cyber sickness, the technical parameters of the virtual reality projection like frame rate, field of view, graphic resolution, bit rate, could be adjusted which leaves open space for new experiments.
